# Epidemiology of injuries in British male ice hockey: A multi‐season prospective cohort study

**DOI:** 10.1002/jeo2.70319

**Published:** 2025-07-02

**Authors:** Ashley Jones, Farzan Kamdin, Declan Eastwood, Gareth Jones

**Affiliations:** ^1^ Musculoskeletal Health and Rehabilitation Research Group School of Health, Leeds Beckett University Leeds UK

**Keywords:** Britain, epidemiology, ice hockey, injury, surveillance

## Abstract

**Purpose:**

The aim of the study was to investigate the incidence, nature and burden of injury in a cohort of male ice hockey players competing in the National Ice Hockey League (NIHL) over two seasons.

**Methods:**

A prospective single‐site cohort study was conducted on 50 players (age: 22.3 ± 3.7) competing over the 2022–2023 and 2023–2024 seasons. All injuries (medical attention and time loss) and illnesses were recorded. Match and training exposure were also recorded. Injury incidence was expressed as injuries per 1000 h of exposure while burden was the number of time loss days per 1000 h of exposure. Prevalence was reported as percentages for: body area, injury type, diagnosis, mechanism, mode of onset and injury episode. One‐variable chi‐squared tests were used to determine if observed values were as expected for each prevalence subcategory.

**Results:**

One hundred and eighty‐two injuries were recorded, of which 26 injuries (26/182) led to time‐loss. Seven illnesses were reported. The injury incidence rate for all injuries was 54.18 (95% confidence interval [CI]: 7.87–62.0)/1000 h, while the time‐loss was 7.74 (95% CI: 2.97–10.71)/1000 h. More injuries were recorded during matches than in training (35.63 [95% CI: 13.81–48.34]/1000 h vs. 0.75 [95% CI: –1.04 to 1.79]/1000 h). Injuries to the head occurred most often (17.6%), although the shoulder was the most burdensome (16.3 severity score). Bone contusions were the most frequent diagnosis (19.8%), although the most days were lost per injury were following muscle contusion injuries (18.3 severity score). Most injuries occurred from (direct and indirect) contact (70.8%) mechanisms, were acute in nature (77.5%) and were classed as first occurrences (86.8%).

**Conclusion:**

The overall incidence of injury was 54.18/1000 h, yet time‐loss injury incidence was much lower at 7.74/1000 h. The most injured body area was the head, and the most injured tissue type was muscle and tendon. Bone contusions were the most common pathology recorded.

**Level of Evidence:**

Level II.

**Abbreviations**
%: percentage
95% CI: 95% confidence intervals
EIHL: Elite Ice Hockey League
h: hours
IIHF: International Ice Hockey Federation
IOC: International Olympic Committee
NIHL: National Ice Hockey League
SCAT: sport concussion assessment tool
SPSS: statistical package for social sciences

## INTRODUCTION

Ice hockey is a physically demanding and intensely competitive contact sport, characterised by intermittent periods of high‐speed game play and player collisions [19]. During match‐play at the professional level, players routinely skate at speeds in excess of 30 miles per hour for bouts of between 30 and 80 s [8, 13]. The competitive season for ice hockey is typically a 7–9 month period during which teams play up to three matches per week [22]. Globally, an estimated 1.5 million male athletes across 79 countries play competitively [7]. In 2023, the number of players registered in Great Britain with the International Ice Hockey Federation (IIHF) was 13,327 (senior players = 6798; under 20 players = 5691; female players = 838) [7]. In Great Britain, ice hockey is organised into multiple tiers, reflecting the professional, semi‐professional and amateur levels of the game. The Elite Ice Hockey League (EIHL) consists of 10 professional teams from England Scotland and Wales [14]. The second tier is the National Ice Hockey League (NIHL) consisting of 11 teams with a blend of professional and semiprofessional players [9]. Below these are regionalised (North and South) leagues that recruit semiprofessional and amateur players [9].

A recent systematic review by Cattaneo et al. estimated that the incidence of injury during matches ranged from 38 injuries/1000 h (lowest) to 88.6 injuries/1000 h (highest) of exposure. In contrast, the incidence of training injuries was lower, ranging from 0.4 injuries/1000 h (lowest) to 2.6 injuries/1000 h (highest) of exposure [4]. The authors concluded that although the risk of injury during training for professional male ice hockey is lower compared to many other sports, the risk increases significantly during competitive play specifically in terms of lower limb injuries [11]. Notably, the studies included within the review did not report data on reinjuries. Failure to precisely categorise injury occurrence impedes the clinical utility of the findings. Capturing reinjury data provides valuable epidemiological insights that inform injury management strategies and return to sport decision making.

To date, no studies in ice hockey have reported injury burden, which is increasingly recognised as a crucial metric for understanding the full impact of injuries and identifying opportunities for prevention [10]. By incorporating injury burden data, researchers and practitioners can gain a clearer picture of the overall impact of injuries on players and develop targeted strategies to mitigate injury risk and enhance player safety [3].

Currently, there is a paucity of injury data in regard of ice hockey players competing in Britain. Inherent cultural differences exist compared to those experienced in Europe and North America and present challenges such as on‐ice availability to practice and/or compete and off‐ice resources such as dedicated medical teams and sport science support [6, 11].

Accurately capturing injury data from players competing in British ice hockey will allow for a comprehensive assessment within this specific context to be made and contribute a valuable insight to the global understanding of the sport's injury profile. Additionally, such information is beneficial locally to clinicians in terms of resource planning and the implementation of targeted injury risk reduction strategies thus promoting player safety [18].

The aim of the study was to investigate the incidence, nature and burden of injury in a cohort of male ice hockey players from a single team competing in the NIHL over two competitive seasons.

## MATERIALS AND METHODS

### Design

A single site, multi‐season prospective cohort study was conducted during the 2022–2023 and 2023–2024 NIHL seasons (September–April).

### Participants recruitment and selection

All players with a professional contract at the enrolled ice hockey club provided written consent to allow their data to be used in the study including reported injuries, illness, match and training exposure throughout the seasons stated. All study data were coded for anonymity. Ethical approval was gained from a university Research Ethics Committee in accordance with the standards of ethics outlined in the Declaration of Helsinki. In total, 50 (*n* = 50) players agreed to participate in the study over the two seasons. Table 1 provides a breakdown of the participant characteristics.

**Table 1:** Player/team characteristics.

	2022–2023 season	2023–2024 season	Overall (two seasons)
Total number of players	26	24	50
Age (years)	22.1 ± 3.7	22.5 ± 3.7	22.3 ± 3.7
Position (goaltender [GT], defenseman [DEF], forward [FWD])	GT—*n* = 2	GT—*n* = 3	GT—*n* = 5
	DEF—*n* = 10	DEF—*n* = 7	DEF—*n* = 17
	FWD—*n* = 14	FWD—*n* = 14	FWD—*n* = 28
Exposure			
Total (h)	1407.0	1952.1	3359.1
Training (h)	1048.4	1615.7	2664.1
Match (h)	358.6	336.4	695.0
Total hours/player	54.1 ± 23.0	81.3 ± 27.7	67.1 ± 28.6
Training hours/player	40.3 ± 15.6	67.3 ± 22.0	53.2 ± 23.1
Match hours/player	13.8 ± 10.8	14.0 ± 10.5	13.9 ± 10.3

*Note*: Values presented as mean ± SD.

### Data collection procedure

Data collection procedures followed the guidelines set out in the International Olympic Committee (IOC) consensus document for studies investigating injury and illness in sport [10].

An injury was defined as tissue damage or other derangement of normal physical function due to participation in sports, resulting from rapid or repetitive transfer of kinetic energy [10]. Both time loss and medical attention injuries were recorded. A time‐loss injury was classified as an injury that results in a player being unable to take a full part in future training or match play for at least one day. For each injury, the following details were recorded: injury recurrence, injury location, injury type, mechanism of injury and mode of onset [10].

Exposure was recorded on a weekly basis using onto a password protected online spreadsheet. A match was defined as organised scheduled play between opposing athletes or teams of athletes, or athlete(s) competing (i) against time and/or (ii) to obtain a score (judged or measured) [10]. Training was defined as physical activities performed by the athlete that are aimed at maintaining or improving their skills, physical condition and/or performance in their sport [10], and was subcategorised as on‐ice and off‐ice training using the following definitions:

On‐ice training—sessions involving the techniques and/or tactics of the ice hockey, performed on the ice, supervised by a coach.
Off‐ice training—sessions solely composed of resistance training and/or conditioning training. These include recovery sessions, rehab, and postrehab transition sessions.

An illness was categorised as a complaint or disorder experienced by an athlete, not related to the injury. Illnesses include health‐related problems in physical (e.g., influenza), mental (e.g., depression), or social well‐being or removal or loss of vital elements (air, water, warmth) [10].

Individual training exposure was captured using attendance registers and a stopwatch to measure training time. Match exposure was captured using the hudl instat analysis software (https://www.hudl.com/en_gb/products/instat), a camera‐based system installed at all match venues hosting NIHL matches. This software provided individual player match minutes for each competitive fixture which allowed for individual player match exposures to be calculated.

Exposure sheets, and injury and illness information were coded for anonymity and returned to the principal investigator via email on a monthly basis. Information was cross‐checked for missing data, ambiguity and the medical team contacted for clarification as necessary.

### Data analysis

Data analysis was conducted using the Statistical Package for Social Sciences (SPSS) version 29 and Microsoft Excel for Windows 365. Descriptive statistics were calculated for the following prevalence subcategories: injury recurrence, injury location, injury type, injury diagnosis mechanism of injury and mode of onset. One variable chi‐squared tests were also used to assess whether observed values met expected values. Injury incidence was calculated using the equation number of injuries per 1000 player hours (h) and was reported for all overall, match and training injuries [21]. Injury burden was expressed as the number of injuries per 1000 days lost. 95% confidence intervals (95% CIs) were also calculated for all incidence and burden rates [10].

## RESULTS

In total, 3359.1 h of exposure (2664.1 training hours and 695.0 match hours) were recorded over the 22–23 and 23–24 seasons (see Table 1). In the overall sample of 50 players, there were 182 injuries of which 86.8% (158/182) were new injuries, 7.7% (14/182) were exacerbations of a current injury and the remaining 5.5% (10/182) were recurrent injuries (5/182 < 2 months from return to play; 5/182 2–12 months from return to play). Overall, 34 players sustained more than one injury (between 2 and 12), while 11 players did not sustain an injury during the season. Of the 182 injuries reported, 26 injuries (14.3%) led to time‐loss totalling 333 days. The remaining 156 (85.7%) injuries led to no time away from hockey training or match play. A total of seven illnesses were reported, leading to a total of 139 days of time‐loss. A full break down of injury and illness rates can be found in Tables 2 and 3, respectively.

**Table 2:** Injury incidence and burden.

	Frequency (%)	Incidence rate (IR) IR/1000 h (CI)	Total days lost	Injury burden TL/1000 h (CI)
2022–2023 and 2023–2024 seasons combined				
Overall	182	54.18 (7.87–62.0)	333	99.13 (10.64–109.78)
TL	26 (14.3)	7.74 (2.97–10.71)		
Non‐TL	156 (85.7)	46.44 (7.28–53.72)		
Match TL	24 (13.1)	34.53 (13.81–48.34)	308	443.16 (49.49–492.65)
Training TL	2 (0.01)	0.75 (−1.04 to 1.79)	25	11.04 (4.32–15.37)
2022–2023 season				
Overall	96	68.23 (13.64–81.870	189	134.32 (19.15–153.47)
TL	16	11.36 (5.57–16.94)		
Non‐TL	80	56.85 (12.45–69.31)		
Match TL	15	41.82 (21.16–62.99)	175	488.0 (72.30–560.31)
Training TL	1	0.95 (−1.86 to 2.82)	14	13.35 (6.99–20.34)
2023–2024 season				
Overall	86	44.05 (9.31–53.36)	144	73.76 (12.04–85.81)
TL	10	5.12 (3.17–8.29)		
Non‐TL	76	38.93 (8.75–47.68)		
Match TL	9	26.75 (17.47–44.23)	133	395.36 (67.19–462.55)
Training TL	1	0.61 (−1.21 to 1.83)	11	6.80 (4.02–10.83)

Abbreviations: %, percentage of total injury number; CI, 95% confidence interval; TL, time‐loss.

**Table 3:** Illness type/aetiology.

	Frequency (%)	Total days lost
Respiratory—infection	2 (28.5)	5
Gastrointestinal—infection	1 (14.2)	4
Musculoskeletal—infection	2 (28.5)	91
Unknown	2 (28.5)	39

Abbreviations: %, percentage of total injury number.

### Incidence of injury

The incidence rate for all injuries across both seasons was 54.18/1000 h. Medical attention loss injuries had an overall incidence rate of 46.44/1000 h whereas time loss injuries occurred at a rate of 7.74/1000 h. Match time loss injuries produced a higher incidence rate than training time loss injuries 35.63 versus 0.75/1000 h.

Overall injury incidence was higher in the 2022–2023 season, compared to the 2023–2024 season, with time loss injuries more than 50% higher (5.12 vs. 11.36/1000 h). Table 2 includes all incidence rates including 95% CI's.

### Body area and type

The head was the most frequently injured body area 17.6% (32/182; 9.53/1000 h), while muscle tendon injuries occurred most often, accounting for 34.1% (62/182; 18.45/1000 h). Bone contusions were diagnosed more than any other injury (19.8%, 36/182; 10.71/1000 h) One‐variable chi‐squared tests found a significant differences between expected and observed values for injury location (*χ* ^2^[16] = 127.549, *p* = <0.001), injury type (*χ* ^2^[15] = 171.055, *p* ≤ 0.001) and injury diagnosis (*χ* ^2^[9] = 237.341, *p* ≤ 0.001). All injuries are subcategorised by body area, tissue type and diagnosis in Table 4.

**Table 4:** Body area, type and diagnosis.

	Frequency (%)	Incidence rate (IR) IR/1000 h (CI)	Total days lost	Injury burden TL/1000 h (CI)
Body area				
Abdomen	2 (1.1)	0.59 (−0.83 to 1.42)	0	0
Ankle	4 (2.2.)	1.19 (−1.17 to 2.36)	19	5.65 (2.54–8.19)
Chest	3 (1.6)	0.89 (−1.01 to 1.90)	0	0
Elbow	5 (2.7)	1.48 (−1.30 to 2.79)	4	1.19 (–1.16 to 2.35)
Foot	6 (3.3)	1.78 (−1.43 to 3.22)	19	5.65 (2.54–8.19)
Forearm	3 (1.6)	0.89 (−1.01 to 1.90)	0	0
Hand	26 (14.3)	7.74 (2.98–10.72)	71	21.13 (4.91–26.05)
Head	32 (17.6)	9.53 (3.30–12.83)	51	15.18 (4.16–19.34)
Hip/Groin	16 (8.7)	4.76 (2.33–7.10)	14	4.16 (2.18–6.35)
Knee	12 (6.6)	3.57 (2.02–5.59)	86	25.60 (5.41–31.01
Lower leg	1 (0.5)	0.30 (−0.58 to 0.88)	0	0
Lumbosacral	1 (0.5)	0.29 (−0.58 to 0.88)	0	0
Neck	6 (3.3)	1.78 (−1.43 to 3.22)	8	2.38 (1.65–4.03)
Shoulder	18 (9.9)	5.36 (2.48–7.83)	49	14.58 (4.09–10.67)
Thigh	19 (10.4)	5.65 (2.54–8.20)	8	2.38 (1.65–4.03)
Thoracic spine	11 (6.0)	3.27 (1.94–5.21)	4	1.19 (–1.16 to 2.35)
Upper arm	3 (1.6)	0.89 (−1.01 to 1.90)	0	0
Wrist	14 (7.7)	4.17 (2.18–6.35)	0	0
Injury type and diagnosis				
Bone	44 (24.2)	13.09 (3.87–16.96)	111	33.04 (6.14–39.19)
Bone contusion	36 (19.8)	10.71 (3.50–14.21)	53	15.77 (4.23–20.02)
Bone stress injury	2 (1.1)	0.59 (−0.82 to 1.42)	0	0
Fracture	6 (3.2)	1.78 (−1.42 to 3.21)	58	17.26 (4.44–21.7)
Brain/spinal cord	4 (2.2)	1.90 (−1.66 to 2.35)	23	6.84 (2.79–9.64)
Concussion	4 (2.2)	1.90 (−1.66 to 2.35)	23	6.84 (2.79–9.64)
Cartilage	6 (3.2)	1.78 (−1.42 to 3.21)	4	1.19 (–1.16 to 2.35)
Articular cartilage	1 (0.5)	0.29 (−0.58 to 0.88)	0	0
Bursitis	5 (2.7)	1.48 (−1.34 to 2.79)	4	1.19 (–1.16 to 2.35)
Internal organs	1 (0.5)	0.29 (−0.58 to 0.88)	0	0
Ligament/Joint capsule	25 (13.7)	7.44 (2.91 to 10.35)	76	22.62 (5.08–27.71)
Ligament sprain	24 (13.2)	7.14 (2.85–10.00)	76	22.62 (5.08–27.71)
Nonspecific	1 (0.5)	0.29 (−0.58 to 0.88)	0	0
Muscle/Tendon	62 (34.1)	18.45 (4.59–23.05)	85	25.30 (5.37–30.68)
Muscle contusion	18 (9.9)	5.35 (2.47–7.83)	55	16.37 (4.32–20.70)
Muscle strain	33 (18.1)	9.82 (3.35–13.17)	30	8.93 (3.19–12.12)
Tendinopathy	11 (6.0)	3.27 (1.93–5.20)	0	0
Peripheral nerve	4 (2.2)	1.90 (−1.66 to 2.35)	8	2.38 (1.65–4.03)
Superficial tissues	34 (18.6)	10.21 (3.40–13.52)	26	7.74 (2.97–10.71)
Abrasion	13 (7.1)	3.87 (2.10–5.97)	0	0
Laceration	18 (9.9)	5.35 (2.47–7.83)	14	4.16 (2.18–6.35)
Contusion (superficial)	3 (1.6)	0.89 (−1.01 to 1.90)	12	3.57 (2.02–5.59)
Vessels	2 (1.1)	0.59 (−0.82 to 1.42)	0	0

Abbreviations: %, percentage of total injury number; CI, 95% confidence interval; TL, time‐loss.

### Injury mechanism, mode of onset and episode

Direct contact injuries accounted for 36.2% (66/182, 19.64/1000 h) of all injuries, with a one‐variable chi‐squared test revealing no significant differences between expected and observed values across the injury mechanisms (*χ* ^2^[2] = 1.890, *p* = 0.389). Acute injuries were reported most often (77.5%; 141/182; 41.97/1000 h), with most injuries classified as a first episode (occurrence) (86.8%; 158/182; 47.03/1000 h). One‐variable chi‐squared tests found significant differences between expected and observed values for mode of onset (*χ* ^2^[3] = 274.132, *p* ≤ 0.001) and injury episode (*χ* ^2^[3] = 372.066, *p* ≤ 0.001). All injuries are sub‐categorised by mechanism, mode of onset and injury episode in Table 5.

**Table 5:** Injury mechanism, mode of onset and episode.

	Frequency (%)	Incidence rate (IR) IR/1000 h (CI)	Total days lost	Injury burden TL/1000 h (CI)
Mechanism				
Direct contact	66 (36.2)	19.64 (4.74–24.38)	149	44.35 (7.12–51.47)
Indirect contact	63 (34.6)	18.75 (4.63–23.38)	148	44.05 (7.09–51.15)
Noncontact	53 (29.2)	15.77 (4.24–20.02)	36	10.71 (3.50–14.21)
Mode of onset				
Acute	141 (77.5)	41.97 (6.92–48.90)	305	90.79 (10.19–100.98)
Repetitive—sudden	15 (8.2)	4.46 (2.25–6.72)	4	1.19 (1.16–2.35)
Repetitive—gradual	26 (14.3)	7.74 (2.97–10.71)	24	7.14 (2.85–10.00)
Injury episode				
First injury	158 (86.8)	47.03 (7.33–54.37)	310	92.28 (10.27–102.56)
Re‐injury	14 (7.7)	4.16 (2.18–6.35)	0	0 N/A
Early recurrence (<2 months)	5 (2.7)	1.44 (−1.30 to 2.79)	19	5.65 (2.54–8.19)
Late recurrence (2–12 months)	5 (2.7)	1.44 (−1.30 to 2.79)	4	1.19 (–1.16 to 2.35)

Abbreviations: %, percentage of total injury number; CI, 95% confidence interval; TL, time‐loss.

### Injury burden

In total, 333 days were lost due to injury across the two seasons which accounted for a burden rate of 99.13/1000 h. Of this total, the majority (308 days; 443.16/1000 h) were a consequence of injuries sustained during match play, compared to training (25 days; 11.04/1000 h). More days were lost in the 2022–2023 season compared to the 2023–2024 season (189 vs. 144 days) which led to a higher burden rate when comparing the seasons in isolation (134.32 vs. 73.76/1000 h). All burden rates (including 95% CIs) are displayed in Table 2.

**Figure 1:**
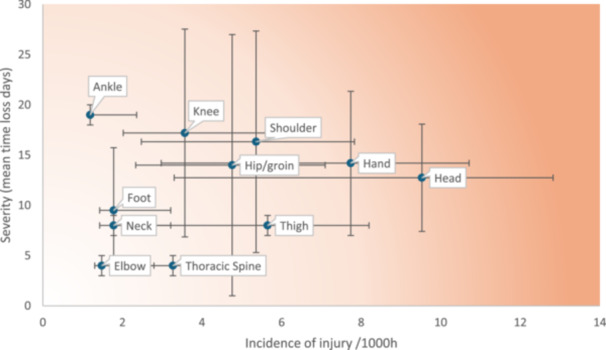
Injury burden expressed as the likelihood (incidence) and consequence (severity) according to injury location. Horizontal lines represent 95% confidence interval (CI) for incidence. Vertical lines represent 95% CI for severity scores.

**Figure 2:**
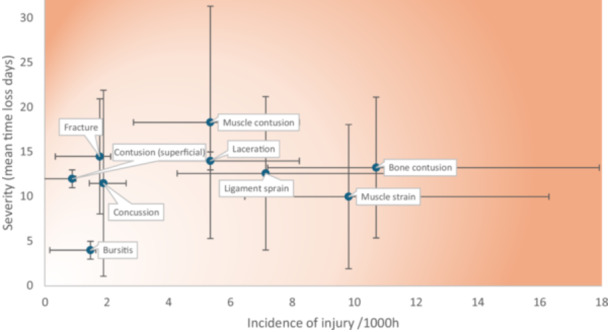
Injury burden expressed as the likelihood (incidence) and consequence (severity) of injury according to injury diagnosis. Horizontal lines represent 95% confidence interval (CI) for incidence. Vertical lines represent 95% CI for severity scores.

When calculating burden by severity, injuries sustained to the ankle (*n* = 19) and knee (*n* = 17.2) caused the highest number of mean days lost. However, the shoulder region was the most burdensome when considering severity and incidence combined (16.3). Muscle contusions were the most burdensome injury type when severity and incidence were combined (*n* = 18.3). Figure 1 shows injury burden expressed as the likelihood (incidence) and consequence (severity) of injury according to location while Figure 2 displays burden according to injury diagnosis.

## DISCUSSION

This is the first study to prospectively report injury incidence, prevalence and burden in a sample of male ice hockey players competing in British ice hockey. A total of 182 injuries were recorded. Of these, only 14% (26/182) resulted in time loss. The overall incidence of injury was 54.18 (95% CI: 7.87–62.0)/1000 h, while time‐loss injuries were recorded at 7.74 (95% CI: 2.97–10.71)/1000 h. More injuries were recorded during matches than in training (35.63 [95% CI: 13.81–48.34]/1000 h vs. 0.75 [95% CI: –1.04 to 1.79]/1000 h). Acute injuries (77.5%) were most often recorded. Injuries were most frequently sustained to the head (17.6%). The knee was found to have the highest burden rate (25.60/1000 h). Injuries to muscle and tendon tissue occurred most often (34.1%) although bone contusions were diagnosed most often (19.8%). The shoulder and muscle contusion injuries were the most burdensome for body region and injury type, respectfully.

In the present study, we found match‐injury incidence to be much higher than training‐injury incidence. This is in broad agreement with a single site cohort study conducted on 94 professional players competing in Japan, where a time‐loss match‐injury incidence of 11.7 (95% CI: 7.5–15.9)/1000 h and a time‐loss training‐injury incidence of 1.1 (95% CI: 0.5–1.7)/1000 h were reported [12]. Further studies across multiple seasons, including male and female collegiate ice hockey players, also confirm this trend of a vastly higher proportion of injuries being sustained during matches relative to training [1, 2]. This is unsurprising and could be explained by the elevated physical contact and psychological pressures athletes experience during competitive ice hockey match, relative to the more controlled and less competitive nature of on‐ice training sessions.

Our study was not the first to obtain injury data from one site over multiple seasons, with several previous studies using this method [12, 19, 24]. Alongside the aforementioned study by Kuzuhara et al. [12], Ornon et al. [19] reported a lower overall injury incidence (5.93 [95% CI: 5.28–6.27]/1000 h) and time loss injuries incidence (2.14 [95% CI: 1.79–2.39]/1000 h), while Suzuki et al. [24] found a much higher overall match injury incidence rate (115.3 [95% CI: 107.4–23.1]/1000 h and 116.8 [95% CI: 109.9–124.7]/1000 h) than us. All three of these previous studies adopted an estimation method for quantifying training and match exposure based on team level reporting, rather than recording individual player exposure. It is plausible to suggest that this may be one reason for the large variances seen across the studies and when compared to our findings. Team level exposure fails to account for individual factors such as player line placement or penalty time, which affect a player's total match minutes. Such discrepancies can lead to the inaccurate reporting of player exposure and the subsequent calculation of injury incidence [23]. The interpretation of injury incidence findings is significantly constrained by methodological inconsistencies. Variations in injury definitions, data collection protocols, exposure calculations, and performance metrics used by authorship teams hinder meaningful comparisons. These discrepancies not only obscure the true nature of injury patterns but also risk producing inaccurate or misleading statistics. To ensure more reliable and comparable results in future research, it is critical that standardised methodologies are adopted across studies, including consistent injury terminology, uniform data collection practices, and standardised measures of exposure and performance. Without these, the validity of cross‐study comparisons remains questionable, and the statistical outcomes may be fundamentally flawed.

The head was the most frequently injured body area (17.6%), followed by the hand (14.3%) and the thigh (10.4%) which concurred with findings from a multisite, 6 year cohort study across National Hockey League (NHL), where head injuries accounted for 16.8% of all injuries [15]. Further work from Suzuki et al. [24] reported wrist/finger injuries accounted for 42.7% of all injuries sustained in training and matches. The categories in this study differ from ours as we subcategorised the wrist and hand separately. While collating the wrist and hand values in the present study increases the total to 22%, this is still significantly lower than the 42.7% presented in the Suzuki et al. [24] study. Differences in injury location may be reflective of the varied levels of contact used across the world. One theory behind variance in contact is differences in rink size, which are said to be smaller rink in North America relative to other countries, leading to more congested play [25]. It is plausible to suggest that due to the high number of North American imports competing in Britain, tactics in British competitions may adopt rougher tactics, compared to the Japanese players included in the study by Suzuki et al. [24].

It was found that bone contusions were the most common pathology (19.8%) followed by muscle strains (18.1%) and ligament sprains (13.2%). This was consistent with findings from the systematic review conducted by Cattaneo et al. [4] where contusions accounted for between 15%–46% of all injuries across 11 primary studies. This is unsurprising due to the nature of the sport, where contact with opposition players, sticks, pucks and advertising boardings are a frequent occurrence. The high number of contact injury mechanisms reported in the present study (70.8%) concur with this theory and also align with previous findings from pooled data reviews [4, 5] where body checking, stick or puck contact were the most frequent contact mechanisms. However, the studies included within these reviews [4, 5] routinely subcategorised injury mechanism by contact type (e.g., puck), which provided a deeper level of analysis than our study.

This is the first to report injury burden in a cohort of ice hockey players using the mean severity relative to individual player exposure in a risk matrix, obtained through medically reported data collection methods (see Figures 1 and 2). Previously, burden has been presented using incidence rates calculated as the number of injuries per player, per year; [16, 17] a method which does not consider individual player exposure. Calculating burden using individual player exposure allows medical staff to better understand which injuries are less frequent but very impactful (in terms of time loss) in a given sport [10].

We found injuries to the head and hand regions demonstrated the highest incidence rates; however, these did not correspond to the greatest mean time‐loss per injury. In contrast, knee and ankle injuries, though less frequent, were associated with greater time loss, indicating higher severity. Shoulder injuries emerged as the region with the highest combination of incidence and severity, suggesting they may represent the most burdensome injuries with respect to player participation and performance. It is important to acknowledge, that the 95% CIs show substantial overlap. Therefore, we recommend medical and coaching staff adopt injury prevention strategies that target both upper and lower limb regions, with the objective of reducing time‐loss injuries and enhancing player availability. As multisite data becomes available using this statistic a better understanding of burden will be revealed.

Medical attention injuries such as contusions and lacerations still require medical intervention to prevent further injury and maintain player availability during training or matches. The high number of immediate trauma injuries reported in our study highlights the need for medical practitioners working in British ice hockey to be competent at administering immediate trauma management strategies such as managing bleeds or wounds. Obtaining these specific skills on accredited prehospital care and/or wound management courses would be beneficial for such staff to both increase player safety and possibly further reduce time‐loss from any subsequent complications that could arise. The high frequency of injuries to the head region also outline the importance of competent concussion assessments, which should be conducted using the most recent sport concussion assessment tool (SCAT) from the Concussion in Sport group [20].

Although conducted over multiple seasons, the authors acknowledge that there are limitations to single‐site cohort studies. Therefore, readers should interpret the findings with a degree of caution, particularly when directly comparing the findings to other published multisite cohort studies. The participants included in this study were a mixture of full time and part time ice hockey players which is common practice throughout teams competing in the NIHL. It is therefore possible that the part time players may have sustained injuries/illness that were resolved between contact days with the club's medical team. It was also impossible to track the volume and type of work‐related activities that were being undertaken away from the ice hockey club which could impacted injury occurrence in this cohort. Future studies should be conducted on full time cohorts where exposure to physical activity and injury/illness can be more closely monitored.

## CONCLUSION

In this cohort of ice hockey players, the overall injury incidence rate was 54.18 (95% CI: 7.87–62.0)/1000 h, yet the time loss injuries accounted for 7.74 (95% CI: 2.97–10.71)/1000 h. More injuries were recorded during matches than in training (35.63 [95% CI: 13.81–48.34]/1000 h vs. 0.75 [95% CI: –1.04 to 1.79]/1000 h). The head was the most commonly injured body area (17.6%), and bone contusions were the most common pathology (19.8%). However, injuries to the shoulder region were the most burdensome, as were muscle contusion injuries. Multisite studies in cohorts competing in British ice hockey competitions are now required.

## AUTHOR CONTRIBUTIONS

**Ashley Jones**: Study conception; literature review; design; manuscript production. **Farzan Kamdin**: Subject recruitment; data collection; manuscript review. **Declan Eastwood**: Subject recruitment; data collection; manuscript review. **Gareth Jones**: Literature review; manuscript production. All authors read and approved the final version of the manuscript.

## CONFLICT OF INTEREST STATEMENT

The authors declare no conflict of interest.

## ETHICS STATEMENT

Ethical approval was granted from the School of Health Research Ethics Committee at Leeds Beckett University (Application Ref: 128951). All participants provided written consent for their data to be used to answer the research question.

## Data Availability

The data that support the findings of this study are available from the corresponding author upon reasonable request.
